# Binding Models of Copper(II) Thiosemicarbazone Complexes with Human Serum Albumin: A Speciation Study

**DOI:** 10.3390/molecules26092711

**Published:** 2021-05-05

**Authors:** Nóra V. May, Attila Jancsó, Éva A. Enyedy

**Affiliations:** 1 Centre for Structural Science, Research Centre for Natural Sciences, Magyar Tudósok Körútja 2, H-1117 Budapest, Hungary; may.nora@ttk.hu; 2 Department of Inorganic and Analytical Chemistry, Interdisciplinary Excellence Centre, University of Szeged, Dóm tér 7, H-6720 Szeged, Hungary; jancso@chem.u-szeged.hu; 3 MTA-SZTE Lendület Functional Metal Complexes Research Group, University of Szeged, Dóm tér 7, H-6720 Szeged, Hungary

**Keywords:** peptide model, albumin binding, EPR spectroscopy, ternary complexes

## Abstract

Copper(II) complexes of thiosemicarbazones (TSCs) often exhibit anticancer properties, and their pharmacokinetic behavior can be affected by their interaction with blood transport proteins. Interaction of copper(II) complexes of an {N,N,S} donor α-*N*-pyridyl TSC (Triapine) and an {O,N,S} donor 2-hydroxybenzaldehyde TSC (STSC) with human serum albumin (HSA) was investigated by UV–visible and electron paramagnetic resonance spectroscopy at physiological pH. Asp-Ala-His-Lys and the monodentate N-methylimidazole were also applied as binding models. Conditional formation constants were determined for the ternary copper(II)-TSC complexes formed with HSA, DAHK, and N-methylimidazole based on the spectral changes of both charge transfer and d-d bands. The neutral N-methylimidazole displays a similar binding affinity to both TSC complexes. The partially negatively charged tetrapeptide binds stronger to the positively charged Triapine complex in comparison to the neutral STSC complex, while the opposite trend was observed for HSA, which demonstrates the limitations of the use of simple ligands to model the protein binding. The studied TSC complexes are able to bind to HSA in a fast process, and the conditional constants suggest that their binding strength is only weak-to-moderate.

## 1. Introduction

**Scheme 1:**
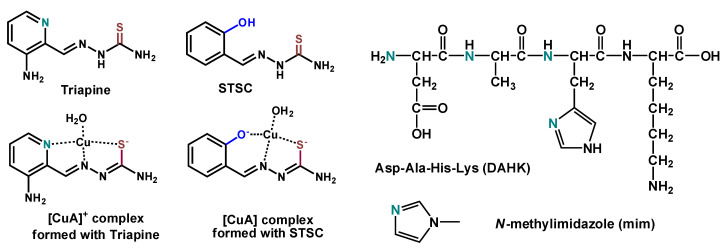
Chemical structures of Triapine, STSC in their neutral forms, and their copper(II) complexes predominating at pH 7.4, and structures of the binding models (DAHK, mim).

Thiosemicarbazones (TSCs) show wide pharmacological versatility including antibacterial, antifungal, antiviral, and antitumor activity. Among them, 3-aminopyridine-2-carboxaldehyde thiosemicarbazone (Triapine, Scheme 1) is the best-known compound [1,2], and it has been tested in more than 30 phase I and II clinical trials in both solid and hematological tumors [3], and a phase III trial is recruiting patients to study the combination of Triapine with cisplatin during radiation [4]. Triapine is administered intravenously, while 4-(2-pyridinyl)-2-(6,7-dihydro-8(5H)-quinolinylid- ene)hydrazide (COTI-2) [5] and di-2-pyridylketone-4-cyclohexyl-4-methyl-3-TSC (DpC) [6] have entered clinical studies as oral drugs. The mechanism of action of anticancer TSCs is often linked to their interaction with endogenous metal ions such as iron and copper [1,2,3]. They can form metal chelates via the {N,S} donor set; however, more diversified binding modes can occur when an additional coordinating group is present in the TSC molecule. Tridentate {N,N,S} coordination mode is realized with α-*N*-pyridyl TSCs (e.g., Triapine), and the {O,N,S} binding motif occurs in complexes of 2-hydroxybenzaldehyde TSCs such as the simplest representative, the salicylaldehyde TSC (STSC, Scheme 1) [3,7,8]. Numerous metal complexes of TSCs were developed and tested as potential anticancer agents, and copper(II) complexes gained a special interest since they often exhibit higher cytotoxicity, compared to their corresponding ligands [9,10,11,12,13,14]. Both Triapine and STSC form highly stable and redox-active complexes with copper(II) [15,16,17,18], and X-ray crystallography studies showed that the metal ion adopts a square planar coordination geometry with the tridentate binding of the TSC ligand and a chlorido co-ligand (or water) [9,18,19]. Based on the solution stability and structural studies, complexes with {N,N,S^−^} and {O^−^,N,S^−^} coordination modes predominate at physiological pH in the case of Triapine and STSC, respectively [15,16]. The co-ligands (Cl^−^ or H_2_O) can be replaced by the donor atoms of endogenous compounds such as proteins in the biofluids, and interaction with human serum albumin (HSA) may have a strong influence on the pharmacokinetic properties of the copper(II) complexes.

HSA is the most abundant protein in the blood with an average concentration of 630 μM, and it greatly augments the transport capacity of serum due to its extraordinary binding affinity toward endogenous and exogenous compounds [20]. Binding to HSA affects the half-life of drugs, and this protein can act as a transport vehicle; thus, HSA is considered as a promising drug delivery system as well [20,21,22]. It should be noted that the microenvironment of malignant and inflamed tissues such as solid tumors often exhibits an increased level of HSA accumulation as a consequence of the enhanced permeability and retention effect [21]. Fairly diverse scenarios are possible considering the binding modes on HSA and reaction rates for metallodrugs [23,24,25]. On the one hand, HSA has three main nonspecific binding pockets located in subdomains IIA, IIA, and IB, where compounds can bind via noncovalent bonds [23,24]. On the other hand, HSA has four partially selective metal-binding sites with well-defined metal preferences [25], and the N-terminal site (also known as ATCUN motif) consisting of the tripeptide sequence Asp-Ala-His can efficiently coordinate to copper(II) in a square planar geometry, with the participation of the N-terminal amine, histidine imidazole and two backbone amides [25]. However, copper(II) complexes with vacant coordination sites can bind to HSA via, e.g., accessible surface imidazole nitrogen donors of histidines as well. Coordination of His242 and Lys199 of HSA to the copper(II) complex of a tridentate Schiff base ligand bearing {O,N,O} donor set was reported by Guo et al. [22], and the adduct formation with the protein increased the anticancer activity of the complex. Yang and Liang developed various copper(II)-α-*N*-pyridinyl TSC-HSA adducts [26,27], which exhibited better selectivity and improved capacity of inhibiting tumor growth in in vivo (mice) tests in comparison to the copper(II)-TSC complexes alone. On the basis of X-ray crystallography analysis of the complex–protein adducts, coordination of His nitrogen donors (His242 and His146) was proved [26,27].

Although the binding affinity of HSA toward copper(II) ions was already reported, and conditional constant was determined by Bal et al. at physiological pH [28], no equilibrium constants are available for the binding of copper(II)-TSC complexes to HSA. In this work, we investigated the interaction of copper(II) complexes of Triapine and STSC (Scheme 1) with HSA and two His-containing simpler ligands by pH-potentiometry, UV–visible (UV–vis) spectrophotometry, and electron paramagnetic resonance (EPR) spectroscopy to reveal the binding strength and rates, namely, the tetrapeptide Asp-Ala-His-Lys (DAHK) consisting of the native HSA sequence for copper(II) binding and the monodentate N-methylimidazole (mim) were selected as binding models (Scheme 1).

## 2. Results and Discussion

### 2.1. Solution Speciation in the Binary Systems

Thiosemicarbazones and their metal complexes are generally characterized by insufficient water solubility, and this is the reason why solution equilibrium studies are often performed in mixtures of water and an organic solvent with a relatively lower dielectric constant. The proton dissociation processes of Triapine and STSC and their complex formation with copper(II) ions were characterized in a 30% (*w/w*) DMSO/H_2_O solvent mixture in our previous works [15,16], and this medium was also used for the speciation studies presented here. Triapine forms high stability mono-ligand [CuHA]^2+^, [CuA]^+^ and [CuA(OH)] complexes (in which A^−^ is the completely deprotonated form of the ligand), and [CuA]^+^ predominates at pH 7.4 characterized by a {N_pyridine_,N,S^−^}{H_2_O} binding mode (Scheme 1). In this species, the negative charge is localized on the sulfur atom due to the thione–thiol tautomeric equilibrium following the loss of the proton from the hydrazonic-*N* atom [15]. Notably, at ligand excess, bis-ligand complexes and the dinuclear species [Cu_2_A_3_]^+^ are also formed [15]. On the contrary, STSC forms exclusively mono complexes and the predominant species is also [CuA] (Scheme 1) at physiological pH; however, in this case, the ligand is coordinated in its dianionic form with an {O^−^,N,S^−^} donor set [16]. It is noteworthy that DMSO weakly coordinates to the copper(II) ions, and as a consequence, the complexes of Triapine, 2-formylpyridine thiosemicarbazone, and their N-terminally dimethylated derivatives were characterized by somewhat higher solution stabilities in pure water in comparison to 30% (*w/w*) DMSO/H_2_O [29,30].

The p*K*_a_ values of DAHK and N-methylimidazole (Scheme 1) and the overall stability constants of their copper(II) complexes were determined by pH-potentiometric titrations (Table 1). DAHK possesses five dissociable protons; however, the dissociation processes overlap resulting in difficulties in the assignment of the p*K*_a_ values to the functional groups; thus, they are considered as macroscopic constants. Based on chemical evidence and data reported for related tri- and tetrapeptides (Asp-Ala-His-NH_2_, Asp-Ala-His-Lys-NH_2_) in pure water [31,32], the two lowest p*K*_a_ values are assigned to the carboxyl groups of Asp and Lys residues, p*K*_3_ belongs to the histidine-nitrogen, while p*K*_4_ and p*K*_5_ to the N-terminal and the side-chain Lys amino groups, respectively. In the copper(II)‒DAHK system formation of mainly mono complexes in different protonation states was found (Table 1), similarly to the speciation model obtained for copper(II)-Asp-Ala-His-Lys-NH_2_ reported by Bal et al. [31]. The C-terminal amide group of Asp-Ala-His-Lys-NH_2_ is not involved in the coordination, and the same probably holds for the C-terminal COOH of DAHK; thus, the speciation models and the overall stability constants can be compared with each other. Bal et al. found four copper(II)-bound nitrogen donors in the square planar [CuH_−2_L]^−^ dominating in a wide pH range including the physiological pH (L^−^ is the completely deprotonated form of the ligand). These are the N-terminal amino group, the amide nitrogen atoms located between Asp and Ala and between Ala and His, and one His nitrogen [31].

**Figure 1:**
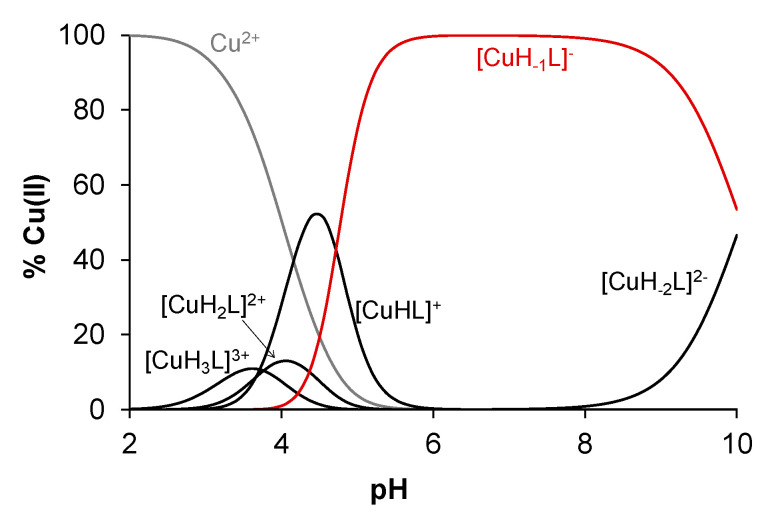
Concentration distribution curves for the copper(II)‒DAHK (1:1) system. {*c*_DAHK_ = *c*_Cu(II)_ = 1 mM; 30% (*w*/*w*) DMSO/H_2_O; *T* = 25 °C; *I* = 0.10 M (KCl)}.

Complexes [CuH_3_L]^3+^, [CuH_2_L]^2+^, [CuHL]^+^, [CuH_−1_L]^−^, and [CuH_−2_L]^2−^ were observed with DAHK in the 30% (*w/w*) DMSO/H_2_O solvent mixture. On the basis of the overall stability constants, concentration distribution curves were calculated (Figure 1), showing the formation of [CuH_3_L]^3+^, [CuH_2_L]^2+^, and [CuHL]^+^ in the acidic pH range, and [CuH_−1_L]^−^ becomes the predominating species at pH > 5.5, while [CuH_−2_L]^2−^ forms in the alkaline medium. Due to the close similarity between the complexation scheme of DAHK and Asp-Ala-His-Lys-NH_2_ [31], the coordination of four nitrogen donors (Asp-N, amide-N of Asp-Ala, amide-N of Ala-His residues, and His-N) can be assumed in the complex [CuH_−1_L]^−^. It should be noted that at ligand excess bis complexes are also present. Additionally, the conditional stability constant for the DAHK complex was also calculated at pH 7.4 in 30% (*w/w*) DMSO/H_2_O, and the obtained value is log*K*’_7.4_ = 14.2, which shows an acceptable agreement with values 13.6 and 13.7 (in pure water) reported for DAHK by isothermal titration calorimetry [32] and for DAHK-NH_2_ obtained by pH-potentiometry [31], respectively.

For N-methylimidazole (Scheme 1), only one p*K*_a_ (6.62) was determined experimentally, attributed to the imidazolium-NH^+^ moiety (Table 1), and it is somewhat lower than the value reported in pure water (p*K*_a_ = 7.05 [33]) since the neutral form of the ligand (L) is better solvated in the presence of DMSO. Complexes with various metal-to-ligand ratios, i.e., [CuL]^2+^, [CuL_2_]^2+^, [CuL_3_]^2+^, and a mixed hydroxido species [CuH_−1_L]^+^ (Table 1), were observed for this monodentate ligand. The calculated EPR parameters are found in Table S1. Notably, the formation of [CuL_4_]^2+^ is suggested (instead of [CuH_−1_L]^+^) based on the recorded frozen solution EPR spectra (Figure S1).

In order to compare the affinity of the ligands toward copper(II), pCu (‒log[Cu(II)]) values were calculated for Triapine, STSC, DAHK, and N-methylimidazole at pH 7.4 (Table 1). These data reveal the strongest copper(II) binding property of the tetradentate DAHK, and the weakest for the monodentate mim, as was expected.

### 2.2. Interaction of the Copper(II)-TSC Complexes with HSA, DAHK, and N-Methylimidazol

**Figure 2:**
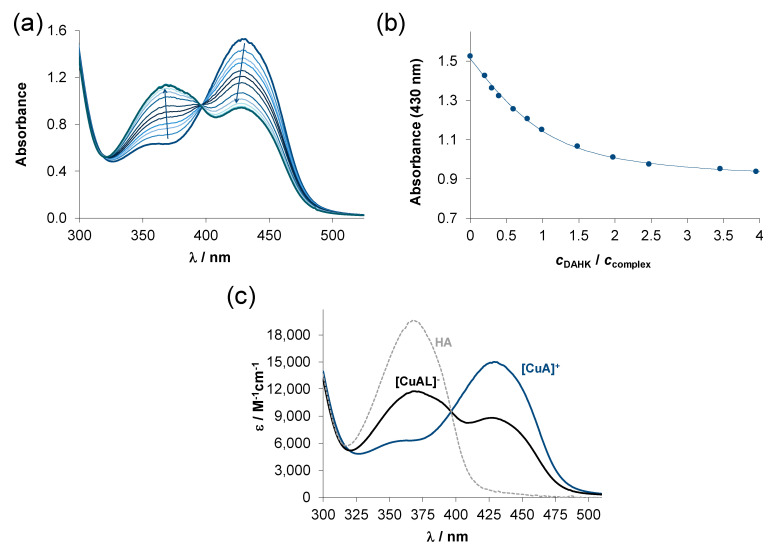
(**a**) UV–vis spectra recorded for the copper(II)‒Triapine‒DAHK (1:1:x) system at pH 7.4 after 4 h equilibration time (x:0–4). (**b**) Absorbance values at 430 nm plotted against the ratio of DAHK and the copper(II)-Triapine complex. (**c**) Molar absorbance spectra of the Triapine complex ([CuA]^+^), the Triapine-DAHK ternary complex ([CuAL]^−^), and the unbound Triapine molecule (HA). {*c*_Cu(II)_ = *c*_Triapine_ = 200 μM, *c*_DAHK_ = 0–800 μM; 30% (*w*/*w*) DMSO/H_2_O; pH = 7.4 (20 mM HEPES); *T* = 25 °C; *I* = 0.10 M (KCl); *ℓ* = 0.5 cm}.

Since the donor atoms occupy only three coordination sites in the copper(II) complexes of Triapine and STSC (Scheme 1), there are vacant sites where the donor atoms of HSA and its simple binding models can coordinate. In order to characterize the binding affinity of the protein, DAHK, and N-methylimidazole to these copper(II)-TSC complexes, conditional binding constants were determined. The copper(II)‒TSC‒DAHK/N-methylimidazole ternary systems were attempted to be studied first by pH-potentiometric titrations applying mM concentration of the components. However, precipitate formation at pH > 6.5 in all systems and slow equilibrium processes with DAHK prevented the execution of titrations. Therefore, formation constants for the ternary complexes were determined at pH 7.4 in 30% (*w*/*w*) DMSO/H_2_O by UV–vis spectrophotometry using lower concentrations, and the spectra were always recorded after a proper equilibration time based on the preliminary time-dependence assays. The reaction between the copper(II)-TSC complex and the oligopeptide DAHK was found to be relatively slow, since at least 30 min was necessary to reach the equilibrium, and thus, a minimum 4 h waiting time was utilized. By increasing the DAHK content of the samples, significant spectral changes were observed (as an example, see spectra for the Triapine complexes in Figure 2a), and the changes ended at ca. four equivalents of the peptide (Figure 2b).

The appearance of clear-cut isobestic points suggests a single equilibrium process, which is most probably the formation of a copper(II)-Triapine-DAHK ternary complex. On the other hand, the absorbance decrease at 430 nm and the increase at 368 nm might be the consequence of the displacement of the original TSC ligand by the stronger copper(II) binder DAHK (see pCu values in Table 1) instead of the formation of a ternary complex. However, the final spectrum recorded for the ternary system at the applied highest excess of DAHK is rather different from the spectrum of the free Triapine (Figure 2c) suggesting the formation of mixed-ligand species (notably, DAHK and its copper(II) complex have no or negligible characteristic absorption bands in the monitored wavelength range (Figure S2)). The S^−^→Cu^2+^ charge transfer (CT) band located at 430 nm displays significant changes indicating a rearrangement in the coordination sphere. Similar but somewhat smaller spectral changes were observed for the STSC complex (Figure S2). Additionally, UV–vis spectra were recorded in the wavelength range of d-d transitions (not shown) using a longer path length (5 cm). The λ_max_ value observed in this wavelength range in the absence of the peptide shifted toward lower wavelength values upon the addition of DAHK (625 nm → 584 nm (Triapine), 583 nm → 540 nm (STSC)).

**Figure 3:**
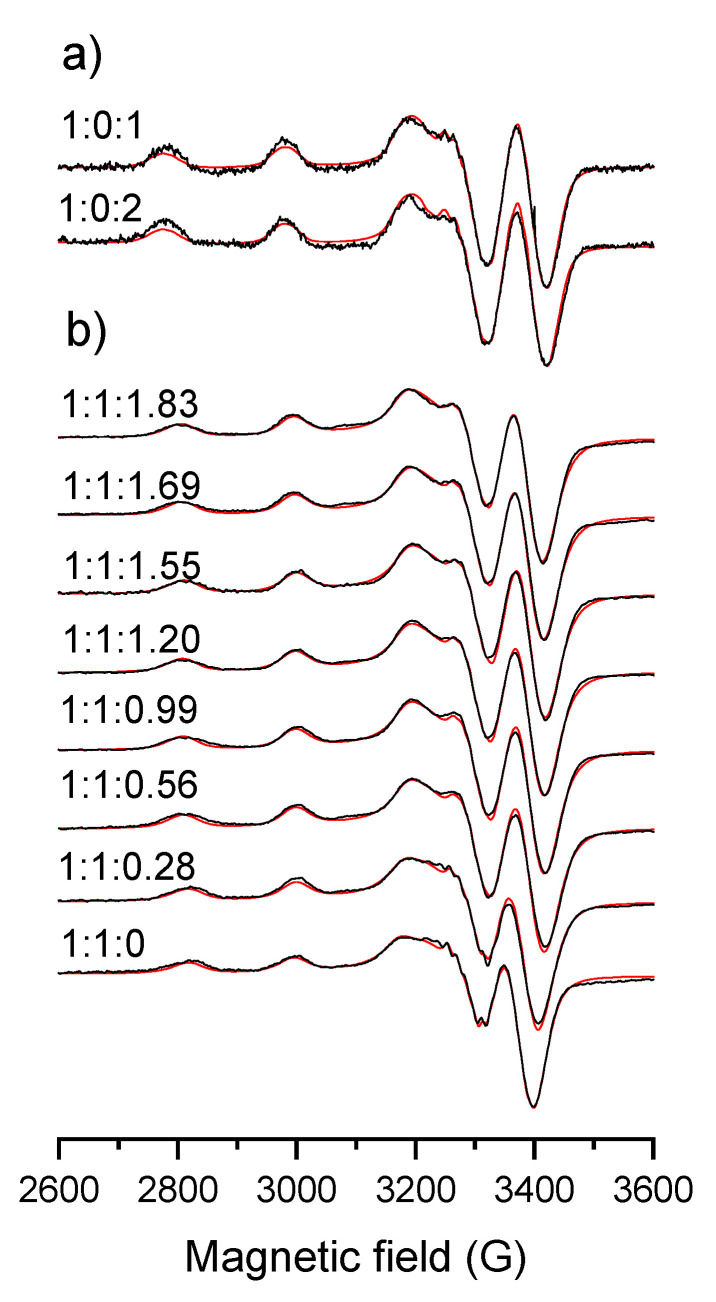
Experimental (black) and simulated (red) anisotropic EPR spectra recorded at 77 K for the (**a**) copper(II)‒DAHK and (**b**) copper(II)‒Triapine‒DAHK systems at pH 7.4 after 4 h equilibration time. Numbers above the spectra indicate the copper(II):Triapine:DAHK ratios. {*c*_Cu(II)_ = *c*_Triapine_ = 495 μM, *c*_DAHK_ = 0–908 μM; 30% (*w*/*w*) DMSO/H_2_O; pH = 7.4 (20 mM HEPES); *I* = 0.10 M (KCl)}.

In order to confirm the formation of ternary complexes anisotropic EPR spectra were recorded for the copper(II)-TSC, copper(II)-DAHK, and the copper(II)-TSC-DAHK ternary systems (for Triapine complexes see Figure 3, and for STSC Figure S3). EPR parameters were calculated for the various species formed in the binary and ternary systems via the deconvolution of the spectra (Table 2). Analysis of these data indicates that the species formed in the ternary systems possess different EPR parameters than the copper(II)-DAHK complex ([CuH_−1_L]^−^), considering especially *A*_x_ and *A*_z_. Upon the interaction of the copper(II)-TSC complexes with DAHK, the *g* factor is decreased in general, while the *A* value is increased suggesting a stronger ligand field due to the coordination of an additional donor atom.

**Figure 4:**
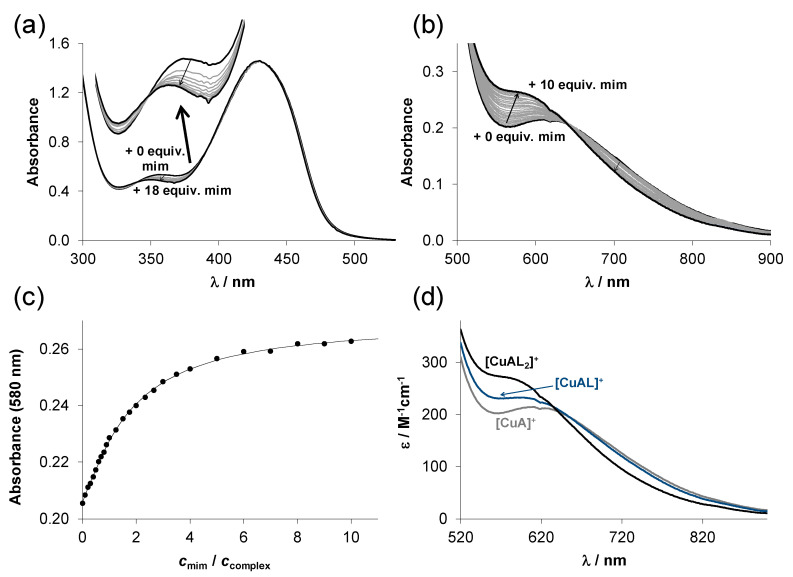
UV–vis spectra recorded for the copper(II)‒Triapine‒N-methylimidazole (mim) system at pH 7.4 after 5 min equilibration time in the wavelength range of the (**a**) CT bands and the (**b**) d-d bands at various equivalents of mim. (**c**) Absorbance values at 580 nm plotted against the ratio of mim and the copper(II)-Triapine complex obtained from the d-d range of the UV–vis spectra. (**d**) Calculated molar absorbance spectra of the Triapine complex ([CuA]^+^), the Triapine-mim ternary complexes ([CuAL]^+^ and [CuAL_2_]^+^). {30% (*w*/*w*) DMSO/H_2_O; pH = 7.4 (20 mM HEPES); *T* = 25 °C; *I* = 0.10 M (KCl); a: *c*_Cu(II)_ = *c*_Triapine_ = 102 μM, *c*_mim_ = 0–1.80 mM; *ℓ* = 1 cm; b: *c*_Cu(II)_ = *c*_Triapine_ = 250 μM, *c*_mim_ = 0–2.50 mM; *ℓ* = 4 cm}.

The interaction of the binary complexes of Triapine and STSC with the monodentate N-methylimidazole was also followed by UV–vis spectrophotometry. The reaction was found to be fairly fast (<5 min), and spectral changes were also observed (Figure 4). However, the changes in the visible range were much more pronounced than in the wavelength range of the CT bands (*c.f.* Figure 4a,b). In order to achieve a complete spectral change a much higher excess of N-methylimidazole was needed (>10) in comparison to DAHK; however, two overlapping processes were observed (Figure 4c).

Based on the recorded UV–vis spectra conditional formation constants (log*K*’) were calculated for the ternary complexes formed with DAHK and N-methylimidazole covering both wavelength ranges (CT and d-d bands) (Table 3) (see representative molar absorbance spectra in Figure 2c and Figure 4d). The constants obtained from the two different wavelength ranges were in good agreement with each other. The obtained results indicate that only one DAHK, compared to two N-methylimidazole ligands, can coordinate to the binary TSC complex. These equilibrium constants reflect the similar binding strength of the neutral N-methylimidazole ligand to both TSC complexes. On the contrary, the binding affinity of the DAHK peptide, which is partially negatively charged at pH 7.4 (32% HL^−^, 60% H_2_L, 8% H_3_L^+^), is somewhat stronger to the positively charged [CuA]^+^ Triapine complex, compared to the neutral [CuA] species of STSC. In these ternary complexes, N-methylimidazole can coordinate via only the imidazole-nitrogen, while DAHK migth coordinate in a bidentate fashion (e.g., via imidazole and terminal amine nitrogen donors forming a macrochelate) (Scheme S1), which leads to the changes of the S^−^ → Cu^2+^ CT band, especially in the copper(II)-Triapine-DAHK complex.

**Figure 5:**
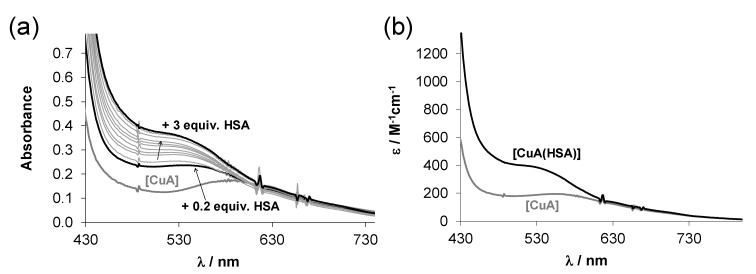
(**a**) Visible spectra recorded for the copper(II)‒STSC‒HSA system at pH 7.4 after 4 h equilibration time in the wavelength range of the d-d bands at various equivalents of HSA and (**b**) the calculated molar absorbance spectra of the binary STSC [CuA], and the ternary [CuA(HSA)] complex. {30% (*w*/*w*) DMSO/H_2_O; pH = 7.4 (20 mM HEPES); *T* = 25 °C; *I* = 0.10 M (KCl); *c*_Cu(II)_ = *c*_TSC_ = 200 μM, *c*_HSA_ = 0–600 μM; *ℓ* = 5 cm}.

Interaction between the two copper(II)-TSC complexes and HSA was followed spectrophotometrically and conditional constants were calculated based on the spectral changes at the CT and d-d bands (Table 3). Representative spectra are shown for the copper(II)‒TSC‒HSA systems in the visible range (Figure 5). Similar to the model compounds, the binding of HSA also results in significant spectral changes. A leveling of the changes could be reached at a lower excess of HSA with STSC (ca. three equivalents), compared to Triapine. It is noteworthy that the addition of various equivalents of the copper(II)-Triapine complex to HSA did not result in measurable changes of the circular dichroism spectra of the protein, suggesting that HSA maintains its α-helical structure upon the interaction (see Figure S4).

**Figure 6:**
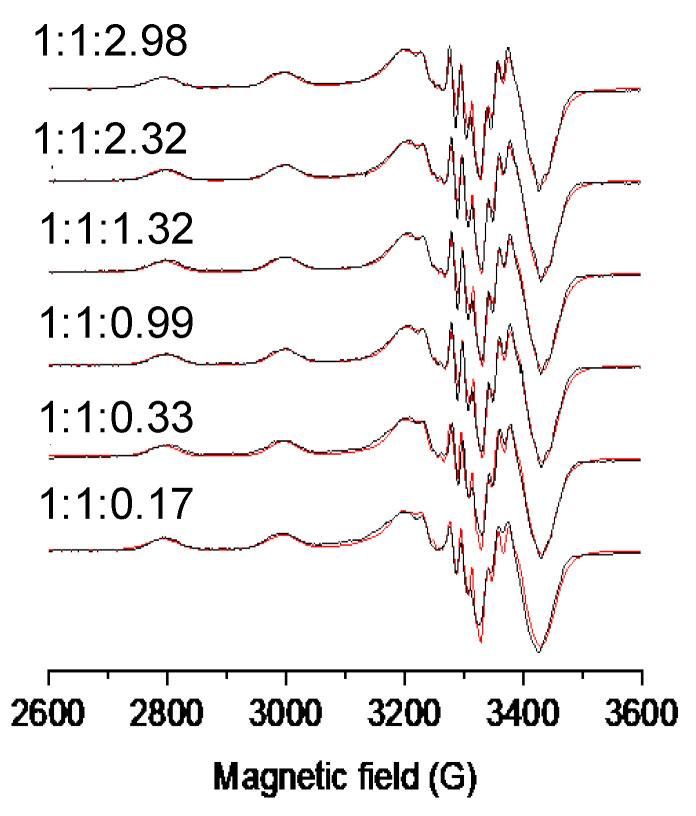
Experimental (black) and simulated (red) anisotropic EPR spectra recorded for the copper(II)‒STSC‒HSA system at pH 7.4 after 4 h equilibration time at 77 K. Numbers above the spectra indicate the copper(II):STSC:HSA ratios. {*c*_Cu(II)_ = *c*_STSC_ = 202 μM, *c*_HSA_ = 0–600 μM; 30% (*w*/*w*) DMSO/H_2_O; pH = 7.4 (20 mM HEPES); *I* = 0.10 M (KCl)}.

EPR spectra were recorded in the copper(II)-STSC system at various excess of HSA (Figure 6), and the EPR parameters (Table 2) calculated for the ternary system differ from those of the binary systems. It should be noted that these parameters reveal differences from the data obtained for the ternary complex of DAHK, suggesting a somewhat altered coordination.

**Figure 7:**
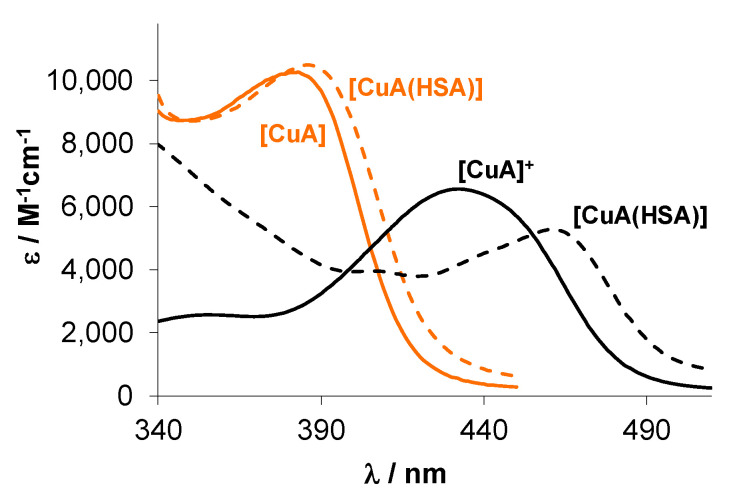
Calculated molar absorbance UV–vis spectra of the binary copper(II)-TSC ([CuA]) and the copper(II)-TSC-HSA ([CuA(HSA)]) ternary complexes in the wavelength range of the CT bands. (Triapine: black lines, STSC: orange lines). {30% (*w*/*w*) DMSO/H_2_O; pH = 7.4 (20 mM HEPES); *T* = 25 °C; *I* = 0.10 M (KCl); *ℓ* = 5 cm}.

The molar absorbance spectra calculated for the ternary adducts of the two TSC complexes formed with HSA (Figure 7) indicate a bathochromic shift of the CT bands upon binding to the protein; however, this type of change is different from those observed for the adducts formed with DAHK and N-methylimidazole. The conditional binding constants reflect a stronger binding of DAHK to the Triapine complex relative to the binding of HSA, while the trend is the opposite with the STSC complex. All these findings suggest that conclusions drawn for the binding modes and strength of HSA on the basis of the results obtained with the simplified model compounds should be considered carefully. Besides the coordinative binding, secondary interactions might play a role as well. The conditional constants determined for the HSA adducts suggest a weak-to-moderate binding affinity of the studied TSC complexes to this protein, namely, ~66% (Triapine) and ~15% (STSC) of the complexes are predicted to be unbound under biologically more relevant conditions (e.g., *c*_HSA_ = 630 μM, *c*_complex_ = 10 μM).

### 2.3. Redox Properties of the Copper(II)-TSC Complexes Affected by HSA and DAHK

**Figure 8:**
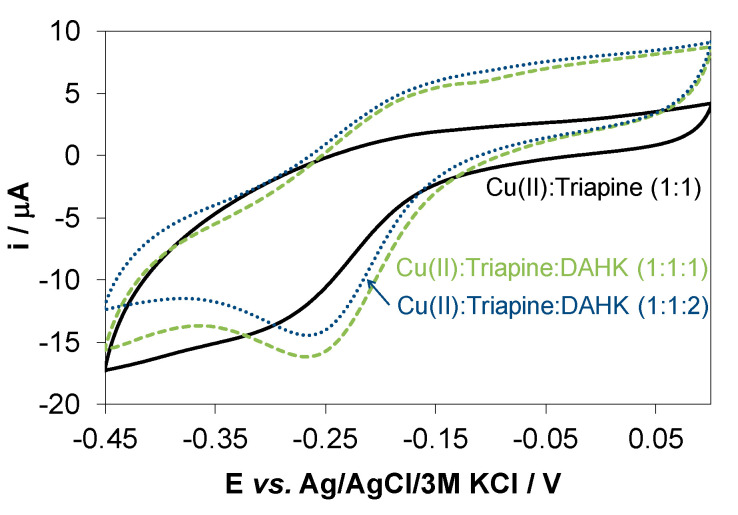
Cyclic voltammograms of the copper(II)‒Triapine (1:1) (solid line) and the copper(II)‒Triapine‒DAHK (1:1:1, dashed line; 1:1:2, dotted line) systems at pH 7.4. {*c*_Cu(II)_ = *c*_Triapine_ = 500 μM, *c*_DAHK_ = 0, 500 or 1000 μM; 30% (*w*/*w*) DMSO/H_2_O; pH = 7.4 (20 mM HEPES); *T* = 25 °C; *I* = 0.10 M (KCl); scan rate: 5 mV/s}.

Since the anticancer activity of the copper(II)-TSC complexes is often related to their redox properties [7,10,29,34], we also investigated whether these features are affected by the binding of HSA (or the model DAHK). Cyclic voltammetric studies were performed for the copper(II)‒Triapine (1:1) system in the absence and presence of DAHK in 30% (*w*/*w*) DMSO/H_2_O at pH 7.4 (Figure 8). Only the position of the cathodic peak could be observed in the voltammograms due to the irreversible nature of the redox processes. The reduction peak potential of the copper(II)-Triapine complex corresponds well to the literature data [18], and a significant shift toward the higher potentials is seen as a result of the coordination of DAHK. It suggests the somewhat stronger oxidizing power of the ternary complex, compared to the binary species.

**Figure 9:**
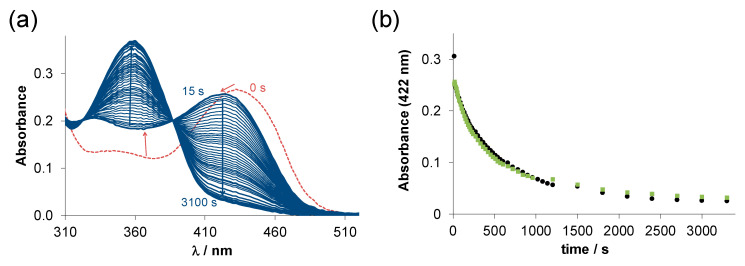
(**a**) Time-dependent changes of the UV–vis absorption spectra of the copper(II)‒Triapine‒HSA (1:1:1) system in the presence of 50 equiv. GSH at pH 7.4 in pure water under argon atmosphere. (**b**) Absorbance changes at 422 nm recorded for the binary copper(II)‒Triapine (●) and for the ternary copper(II)‒Triapine‒HSA (■) systems. {*c*_Cu(II)_ = *c*_Triapine_ = *c*_HSA_ = 25 μM, *c*_GSH_ = 1250 μM; pH = 7.4 (20 mM HEPES); *T* = 25 °C; *I* = 0.10 M (KCl); *ℓ* = 1 cm}.

Investigation of the redox reaction of these copper(II) complexes with physiological reductants can provide more direct information about their ability to react with reducing agents than solely the values of reduction peak potentials. Therefore, the reaction of the copper(II)-Triapine complex in the presence of HSA (and DAHK) with ascorbic acid and glutathione (GSH) was followed spectrophotometrically under anaerobic conditions. The copper(II)-Triapine complex alone was already studied in one of our previous works [30,34]. Ascorbic acid, which is a weaker reducing agent than GSH, was not able to reduce the copper(II) complex either in the absence or in the presence of HSA. On the contrary, the addition of GSH resulted in significant spectral changes (Figure 9a).

For the copper(II)‒Triapine system, it was reported that a ternary Cu(II)-Triapine-GSH complex is formed in the first step after mixing the reactants. Then, the free TSC ligand appears in the solution as a consequence of the dissociation of the generated copper(I) complex since copper(I) tends to form a stable complex with GSH, which is present in a high excess, compared to the TSC. In the presence of one equivalent HSA, the initial spectrum differs from that of the copper(II)-Triapine complex due to the formation of the ternary adduct. Moreover, upon the addition of GSH, the spectrum becomes identical to that observed without the protein. The rate of the subsequent redox reaction is also very similar (Figure 9b). The same phenomenon was observed in the presence of DAHK. Accordingly, it can be concluded that the redox potential is changed due to the ternary complex formation; however, the donor atoms of the protein (or DAHK) can be replaced by other endogenous ligands such as GSH.

## 3. Materials and Methods

### 3.1. Chemicals

Triapine, STSC, HEPES, GSH, ascorbic acid, mim, and HSA (A8763, essentially globulin free) were purchased from Sigma-Aldrich (Hungary), while KCl, HCl, KOH, and DMSO were from VWR (Hungary). DAHK was obtained from GenScript (the Netherlands). The concentration of the stock solution of CuCl_2_ was determined by complexometry using a standard solution of EDTA (VWR, Hungary). The strong acid content of the metal stock solutions was determined by pH-potentiometric titrations. All solvents were of analytical grade and used without further purification. Milli-Q water was used for sample preparations.

### 3.2. pH-Potentiometry

The pH-potentiometric measurements for the determination of the proton dissociation constants of DAHK, mim, Triapine, and STSC, and the overall stability constants of the copper(II) complexes were carried out at 25.0 ± 0.1 °C in a 30% (*w*/*w*) DMSO/H_2_O solvent mixture. The titrations were performed in the pH range of 2.0–12.5 at an ionic strength of 0.10 M (KCl) with a carbonate-free KOH solution of known concentration (0.10 M). The concentrations of the base and the HCl were determined by pH-potentiometric titrations. An Orion 710A pH-meter equipped with a Metrohm combined glass electrode (type 6.0234.100) and a Metrohm 665 Dosimat autoburette were used for the titrations. The electrode system was calibrated to the pH = −log[H^+^] scale in the DMSO/H_2_O medium by means of blank titrations (HCl vs. KOH), similar to the method suggested by Irving et al. [35] in pure aqueous solutions. The average water ionization constant, p*K*_w_, was 14.54 ± 0.05, which corresponds well to the literature data [15,16]. The initial volume of the samples was 5.00 or 10.0 mL. The ligand concentrations were 1 × 10^−3^ (DAHK) or 5 × 10^−3^ M (mim). The copper(II):ligand ratios were varied between 1:1–1:2 (DAHK), or 1:1–1:10 (mim) in the binary systems, while 1:1:1, 1:1:1.5, and 1:1:2 copper(II):TSC:model ligand ratios were applied in the ternary systems at 1 × 10^−3^ M copper(II) and TSC concentrations. Samples were deoxygenated by bubbling purified argon through the solutions for approximately 10 min prior to the measurements. Argon was also passing over the solutions during the titrations. The exact concentrations of the ligand stock solutions, together with the proton dissociation constants, were determined by pH-potentiometric titrations with the use of the computer program HYPERQUAD [36]. It was also utilized to establish the stoichiometry of the complexes and to calculate the formation constants (*β*(M_p_L_q_H_r_)). *β*(M_p_L_q_H_r_) is defined for the general equilibrium pM + qL + rH **⇌** M_p_L_q_H_r_, *β* (M_p_L_q_H_r_) = [M_p_L_q_H_r_]/[M]^p^[L]^q^[H]^r^, where M denotes the metal ion and L the completely deprotonated ligand. In all calculations, titration data were used exclusively from experiments in which no precipitate was visible during the titrations.

### 3.3. UV–Vis Spectrophotometry

A Thermo Scientific Evolution 220 spectrophotometer was used to record the UV–vis spectra. Equilibrium constants and the molar absorbance spectra of the individual species were calculated with the computer program PSEQUAD [37]. Spectrophotometric titrations were performed when the reaction was found to be fast or batch samples were used when longer time was needed to reach the equilibrium (>10 min). The samples contained 1.0 × 10^−4^ M copper(II) for monitoring the CT bands, while 1 × 10^−3^ or 0.5 × 10^−3^ M for the d-d bands. The copper(II):TSC ratio was always 1:1, and HSA, DAHK, or mim was added at various concentrations (up to 10-fold excess). The measurements were carried out at pH 7.40 (2 × 10^−2^ or 5 × 10^−2^ M HEPES) at 25.0 ± 0.1 °C in DMSO:water 30:70 (*w*/*w*) at an ionic strength of 0.10 M (KCl).

The redox reactions of the Cu(II)-TSC complexes with GSH and ascorbic acid in the presence or absence of HSA, DAHK, or mim were studied in pure water at 25.0 ± 0.1 °C on a Hewlett Packard 8452A diode array spectrophotometer using a special, tightly closed tandem cuvette (Hellma Tandem Cell, 238-QS). The reactants were separated until the reaction was triggered. Both isolated pockets of the cuvette were completely deoxygenated by a stream of argon bubbling through the sample for 10 min before mixing the reactants. Spectra were recorded before and immediately after the mixing, and spectral changes were followed until no further absorbance change was observed. One of the isolated pockets contained the reducing agent, while the other contained the metal complex, and their final concentrations after mixing were 1.25 × 10^−3^ M and 2.5 × 10^−5^ M, respectively. The pH of each solution was adjusted to 7.40 by a 50 mM HEPES buffer and an ionic strength of 0.10 M (KCl) was applied. The stock solutions of the reducing agents and the complexes were freshly prepared every day.

### 3.4. EPR Spectroscopy

All EPR spectra were recorded with a BRUKER EleXsys E500 spectrometer (microwave frequency 9.81 GHz, microwave power 10 mW, modulation amplitude 5 G, modulation frequency 100 kHz). The stock solution contained 5 × 10^−4^ or 2 × 10^−4^ M CuCl_2_ with one equivalent of TSC ligand and 0–3 equivalents of HSA or DAHK in 30% (*w*/*w*) DMSO/H_2_O at an ionic strength of 0.10 M (KCl) at pH 7.4 (2 × 10^−2^ M HEPES). The samples were measured in a Dewar containing liquid nitrogen (at 77 K). The anisotropic spectra were analyzed individually with the EPR program [38], which gives the anisotropic EPR parameters (*g*_x_, *g*_y_, *g*_z_, *A*_x_^Cu^, *A*_y_^Cu^, *A*_z_^Cu^, *A*_x_^N^, *A*_y_^N^, *A*_z_^N^, and the orientation-dependent linewidth parameters). Since a natural CuCl_2_ was used for the measurements, the spectra were calculated as the weighted sum of the spectra of ^63^Cu and ^65^Cu according to their natural abundances. The copper and nitrogen coupling constants and the relaxation parameters were obtained in field units (Gauss = 10^−4^ T).

### 3.5. Cyclic Voltammetry

Cyclic voltammograms of the copper(II) complexes in 30–70% (*w*/*w*) DMSO/0.02 M HEPES (pH = 7.4) solution containing 5 × 10^−4^ M CuCl_2_, 5 × 10^−4^ M Triapine and 0, 5 × 10^−4^ or 1 × 10^−3^ M DAHK were determined at 25.0 ± 0.1 °C. Ionic strength was 0.10 M (KCl). Measurements were performed on a conventional three-electrode system under nitrogen atmosphere using an Autolab PGSTAT 204 potentiostat/galvanostat monitored by Metrohm’s Nova software. Samples were purged with argon for 15 min before recording the cyclic voltammograms. A platinum electrode was used as the working and auxiliary electrode and Ag/AgCl/3 M KCl as a reference electrode. The electrochemical system was calibrated with an aqueous solution of K_3_[Fe(CN)_6_] (*E*_1/2_ = +0.386 V vs. NHE).

## 4. Conclusions

Interaction of copper(II) complexes of Triapine and STSC with HSA and its binding models DAHK and N-methylimidazole was monitored by UV–vis and EPR spectroscopic methods at physiological pH. It was found that DAHK has a higher affinity toward copper(II) than the tridentate thiosemicarbazones at pH 7.4 due to the strong coordination of four nitrogen donor atoms, while the monodentate N-methylimidazole was found to be the weakest copper(II) binder. DAHK reacts with the TSC complexes much slower than the protein and N-methylimidazole. Formation of ternary copper(II)-TSC-DAHK complexes resulted in significant changes of the S^−^ → Cu^2+^ charge transfer band suggesting the rearrangement of the coordination sphere. The calculated anisotropic EPR parameters showed a stronger ligand field upon the coordination of DAHK to the copper(II)-TSC complexes. Conditional formation constants were calculated for the ternary complexes formed with DAHK and N-methylimidazole from the spectral changes of the CT and d-d bands. These equilibrium constants revealed the similar binding strength of the neutral N-methylimidazole to both TSC complexes, while the partially negatively charged DAHK exhibits a stronger affinity toward the positively charged Triapine complex in comparison to the neutral STSC complex. The binding of DAHK resulted in somewhat more positive cathodic redox potentials, although the ternary complexes formed with DAHK (and HSA) could be reduced by GSH with the same rate and extent as the parent TSC complexes. Formation of ternary copper(II)-TSC-HSA complexes was proved by UV–vis and EPR spectroscopy, and the spectral characteristics suggest different binding modes of HSA, compared to the tetrapeptide. The binding of HSA to the STSC complex was found to be stronger than to the Triapine complex, and this trend is the opposite of that observed for DAHK. This observation highlights that simplified binding models should be used with special and careful consideration since secondary interactions may also play role in the protein binding of these copper(II) complexes besides the coordination bonds. Based on the conditional constants, the title TSC complexes bind to HSA with weak-to-moderate strength, suggesting a relatively lower fraction of the complexes bound to the protein in the blood serum under biologically relevant conditions.

## Figures and Tables

**Table 1:** p*K*_a_ values of DAHK and mim, overall stability constants (log*β*) of their copper(II) complexes in 30% (*w*/*w*) DMSO/H_2_O,^a^ and pCu values ^b^ (calculated at pH 7.4 at *c*_Cu(II)_ = *c*_ligand_ = 100 μM). {*T* = 25 ^○^C, *I* = 0.10 M (KCl)}.

DAHK		Cu(II)‒DAHK		mim		Cu(II)‒mim	
p*K*_1_	2.82(5)	Log*β* [CuH_3_L]^3+^	27.15(14)	p*K*_1_	6.62(1)	log*β* [CuL]^2+^	4.76(4)
p*K*_2_	3.59(4)	log*β* [CuH_2_L]^2+^	23.39(9)			log*β* [CuL_2_]^2+^	7.84(9)
p*K*_3_	6.40(3)	log*β* [CuHL]^+^	19.73(3)			log*β* [CuL_3_]^2+^	11.45(5)
p*K*_4_	7.69(2)	log*β* [CuH_−1_L]^−^	10.30(3)			log*β* [CuH_−1_L_3_]^+^	3.68(7)
p*K*_5_	10.44(2)	log*β* [CuH_−2_L]^2−^	0.24(5)				
		log*β* [CuHL_2_]^−^	31.47(5)				
		log*β* [CuL_2_]^2−^	23.76(7)				
pCu = 11.12				pCu(II) = 4.44			

^a^ Uncertainties (SD) of the last digits are in parenthesis. ^b^ pCu calculated for Triapine: 7.79, for STSC: 9.40 (in 30% (*w*/*w*) DMSO/H_2_O, 0.1 M KCl) and for HSA: 8.00 (in 0.1 M NaCl, pure water) 7.70 (in 0.1 M NaCl, 0.1 M 2-[4-(2-hydroxyethyl)piperazin-1-yl]ethanesulfonic acid (HEPES) buffered pure water) based on data in Refs. [15,16,28].

**Table 2:** Anisotropic EPR parameters calculated for the various species formed in the binary copper(II)‒Triapine/STSC/DAHK and ternary copper(II)‒Triapine/STSC (A)‒DAHK (L)/HSA systems in 30% (*w*/*w*) DMSO/H_2_O at pH 7.4 (20 mM HEPES).^a^ {*I* = 0.10 M (KCl)}.

Cu(II) Complexes	Triapine [CuA]^+^	Triapine-DAHK [CuAL]^−^	STSC [CuA]	STSC-DAHK [CuAL]^2−^	STSC-HSA [CuA(HSA)]	DAHK [CuH_−1_L]^−^
*g*_x_	2.0278	2.0259	2.0408	2.0361	2.0225	2.0276
*g*_y_	2.0579	2.0547	2.0503	2.0472	2.0444	2.0545
*g*_z_	2.1843	2.1798	2.2069	2.1853	2.1709	2.1801
*g*_0,calc_^b^	2.0900 ^c^	2.0868	2.0993 ^d^	2.0895	2.0793	2.0874
*A*_x_ (G)	30.6	31.6	22.7	36.6	31.1	8.6
*A*_y_ (G)	34.5	38.2	18.2	34.5	41.5	32.3
*A*_z_ (G)	173.4	185.5	180.7	190.0	198.9	200.2
*a*_x_ (G)	12.6/15.5	9.1/14.2	20.0	12.3/16.3	5.5/18.0	13.2/19.2
*a*_x_ (G)	16.6/10.0	14.7/12.7	15.7	19.3/18.8	20.5/9.5	15.5/9.9
*a*_x_ (G)	8.0/8.1	8.0/10.8	12.0	8.0/13.0	14.5/12.6	16.1/10.0

^a^ The experimental errors are ±0.001 for *g*, ± 1 G for A and *a*_N_ tensor values. ^b^ Isotropic values of the g tensor were calculated via equation: *g*_0_ = (*g*_x_ + *g*_y_ + *g*_z_)/3. ^c^ *g*_0_ = 2.0958 (obtained from room temperature measurement) [15]. ^d^ *g*_0_ = 2.0945 (room temperature) [16].

**Table 3:** Conditional stability constants (log*K*’) for the formation of ternary copper(II)‒STSC‒DAHK/mim/HSA complexes in 30% (*w*/*w*) DMSO/H_2_O at pH 7.4 (20 mM HEPES) determined by UV–vis spectrophotometric measurements. In systems of DAHK and HSA 4 h, while with min only 5 min equilibration time was applied. Uncertainties (SD) of the last digits are in parenthesis. {*T* = 25 °C; *I* = 0.10 M (KCl)}.

Triapine				STSC			
ligand L	band	constant(s)		ligand L	band	constant(s)	
DAHK	CT	log*K*’	4.40(5)	DAHK	CT	log*K*’	3.17(6)
DAHK	d-d	log*K*’	4.41(9)	DAHK	d-d	log*K*’	2.94(6)
mim	CT	log*K_1_*’ log*K_2_*’	4.89(8) 3.38(6)	mim	CT	log*K_1_*’ log*K_2_*’	4.74(4) 3.28(4)
mim	d-d	log*K_1_*’ log*K_2_*’	4.72(6) 3.33(6)	HSA	CT	log*K*’	4.04(7)
HSA	CT	log*K*’	2.91(11)		d-d	log*K*’	4.08(3)
HSA	d-d	log*K*’	2.88(5)				

## Data Availability

Not applicable.

## Supplementary Materials

Figure S1: Simulated EPR spectra calculated for the copper(II) complexes of mim with the parameters given in Table S1; and measured and calculated frozen solution EPR spectra, Figure S2: UV–vis spectra recorded for the copper(II)-STSC-DAHK system at pH 7.4, Figure S3: Simulated EPR spectra calculated for species formed in the copper(II)-STSC-HSA system with parameters given in Table 2; and measured and calculated frozen solution EPR spectra, Figure S4: Circular dichroism spectra recorded for HSA in the absence and in the presence of the copper(II)-Triapine complex, Scheme S1: Possible structures for the [CuAL] ternary complexes, Table S1: EPR parameters of the components obtained in Cu-mim solutions.

## Sample Availability

The samples of compounds are not available from authors.
