# Supplementary material for: Binding Models of Copper(II) Thiosemicarbazone Complexes with Human Serum Albumin: A Speciation Study
Source: Molecules. 2021 May 5;26(9):2711. doi: 10.3390/molecules26092711 (PMC8125041; doi:10.3390/molecules26092711)
Supplement: Supplementary file 1 [file molecules-26-02711-s001.zip › molecules-1199066-supplementary.pdf]

# Supporting Information for

## Binding models of copper(II) thiosemicarbazone complexes with human serum albumin: a speciation study

**Nóra V. May [1], Attila Jancsó [2] and Éva A. Enyedy [2,3]***

[1] Centre for Structural Science, Research Centre for Natural Sciences, Magyar tudósok körútja 2, H-1117 Budapest, Hungary
[2] Department of Inorganic and Analytical Chemistry, Interdisciplinary Excellence Centre, University of Szeged, Dóm tér 7, H-6720 Szeged, Hungary
[3] MTA-SZTE Lendület Functional Metal Complexes Research Group, University of Szeged, Dóm tér 7, H-6720 Szeged, Hungary

**Table S1.** EPR parameters of the components obtained in Cu-mim solutions[a]

| Component | $g_x$ | $g_y$ | $g_z$ | $A_x$/G | $A_z$/G | $A_z$/G | $a_x^N$/G | $a_y^N$/G | $a_z^N$/G | $g_{0,calc}$[b] |
|---|---|---|---|---|---|---|---|---|---|---|
| $[CuL]^{2+}$ | 2.051 | 2.058 | 2.315 | 17.2 | 17.4 | 149.0 | 11.0 | 8.7 | 10.0 | 2.1411 |
| $[CuL_2]^{2+}$ | 2.046 | 2.062 | 2.288 | 26.3 | 18.7 | 158.9 | 15.3 | 15.1 | 11.0 | 2.1320 |
| | | | | | | | 12.0 | 15.9 | 10.0 | |
| $[CuL_3]^{2+}$ | 2.047 | 2.062 | 2.284 | 28.7 | 20.7 | 160.5 | 15.6 | 15.9 | 13.3 | 2.1309 |
| | | | | | | | 15.6 | 15.9 | 13.3 | |
| | | | | | | | 14.5 | 15.7 | 7.0 | |
| $[CuL_4]^{2+}$ | 2.049 | 2.050 | 2.253 | 18.0 | 19.3 | 182.9 | 14.0 | 16.3 | 13.3 | 2.1174 |
| | | | | | | | 14.0 | 16.3 | 13.3 | |
| | | | | | | | 16.9 | 12.8 | 7.0 | |
| | | | | | | | 16.9 | 12.8 | 7.0 | |

[a] The experimental error were ±0.001 for *g,* ± 1 G for *A* and $a_N$ tenzor values. [b] Isotropic values of the *g* tensor were calculated via equation: $g_0 = (g_x + g_y + g_z)/3$.

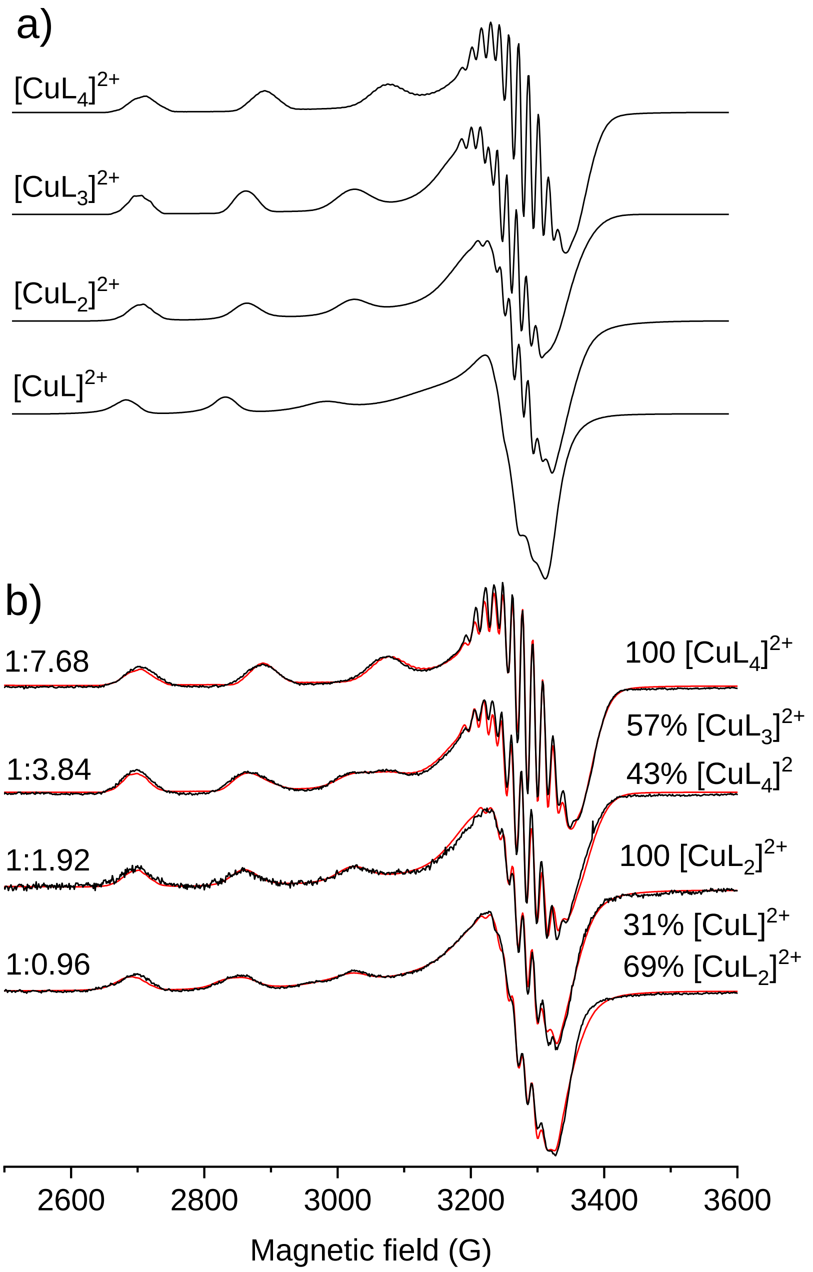


**Figure S1. a**) Simulated EPR spectra calculated for the copper(II) complexes of mim with the parameters given in Table S1. **b**) Measured (black) and calculated (red) frozen solution EPR spectra recorded at $c_{Cu(II)}$ = 2.18×10$^{-4}$ M, numbers above the spectra indicate the different copper(II) - mim concentration ratios.

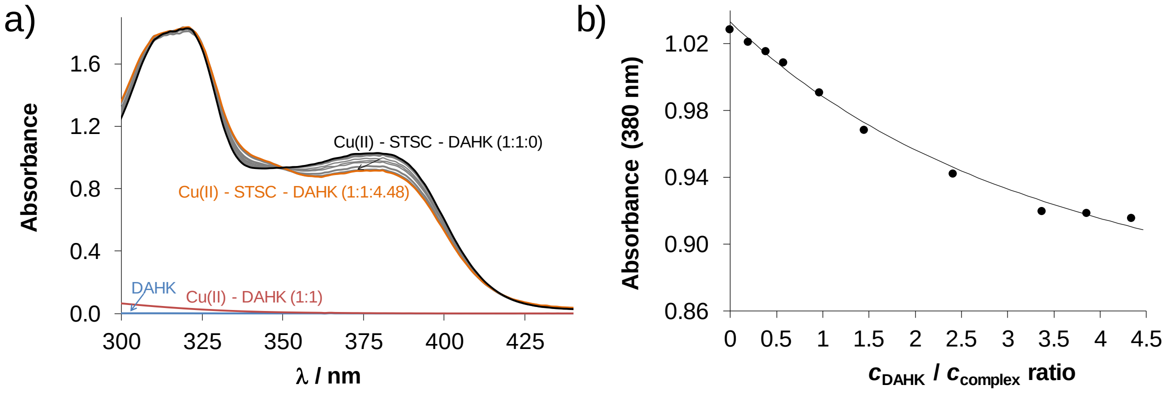


**Figure S2.** **a**) UV-vis spectra recorded for the copper(II) – STSC – DAHK (1:1:x) system at pH 7.4 after 4 h equilibration time (x: 0 – 4.5). **b**) Absorbance values at 380 nm plotted against the ratio of DAHK and the copper(II)-STSC complex). {$c_{Cu(II)}$ = $c_{STSC}$ = 200 µM, $c_{DAHK}$ = 0–896 µM; 30% (w/w) DMSO/$H_2O$; pH = 7.4 (20 mM HEPES); $T$ = 25 ˚C; $I$ = 0.10 M (KCl); $\ell$ = 0.5 cm}

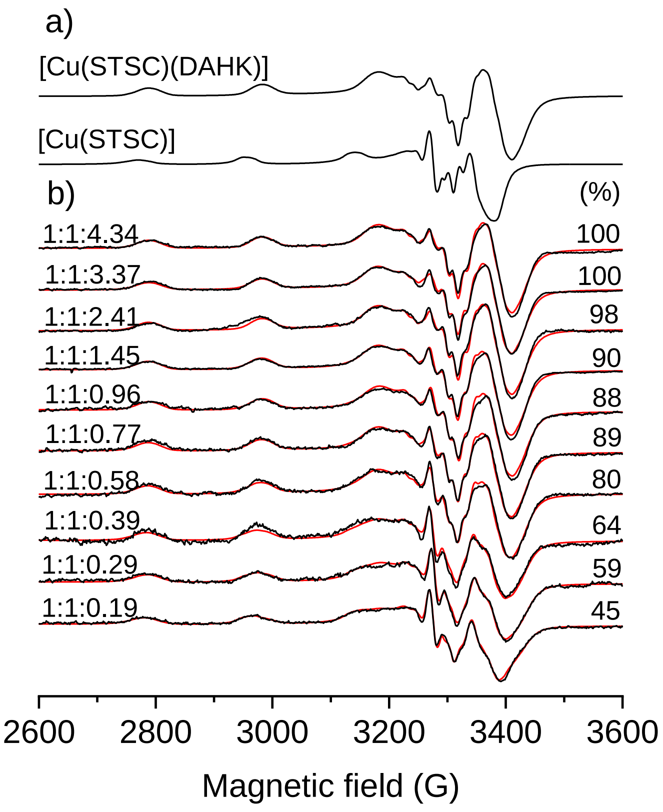


**Figure S3.** **a**) Simulated EPR spectra calculated for species formed in the copper(II) – STSC – HSA system with parameters given in Table 2. **b**) Measured (black) and calculated (red) frozen solution EPR spectra recorded for the same system; numbers above the spectra indicate the different copper(II)-to-STSC-to-DAHK ratios. The ratio (%) refers to the fraction of [Cu(STSC)(DAHK)] component taken into account for the simulation of the spectra.

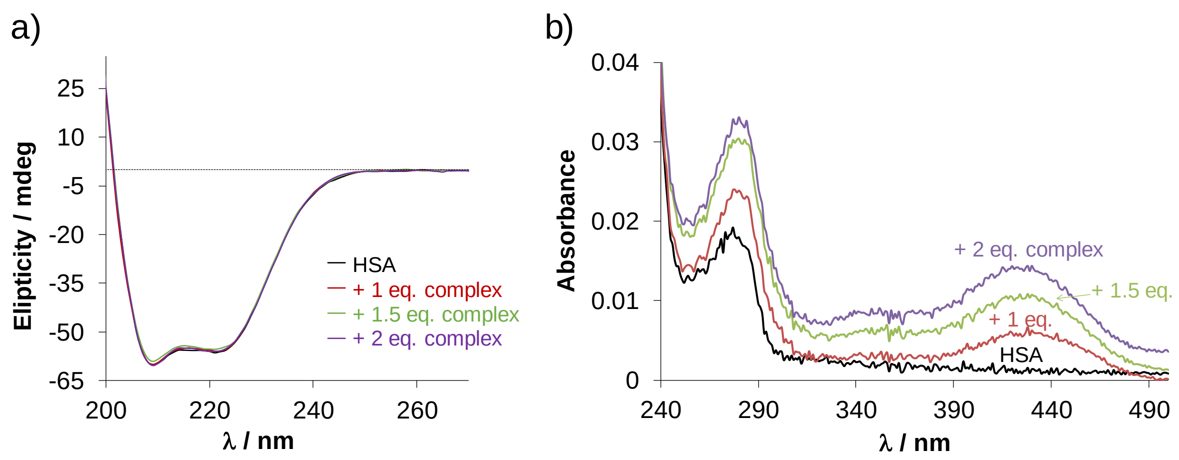


**Figure S4. a**) Circular dichroism spectra recorded for HSA alone and in the presence of various equivalents of the copper(II)-Triapine complex in pure water at pH 7.4, and **b**) the corresponding UV-vis spectra. The circular dichroism spectra were recorded on a JASCO J-1500 instrument. {$c_{HSA}$ = 5 μM; $T$ = 25 ˚C; $I$ = 0.10 M (KCl); $\ell$ = 1 cm}

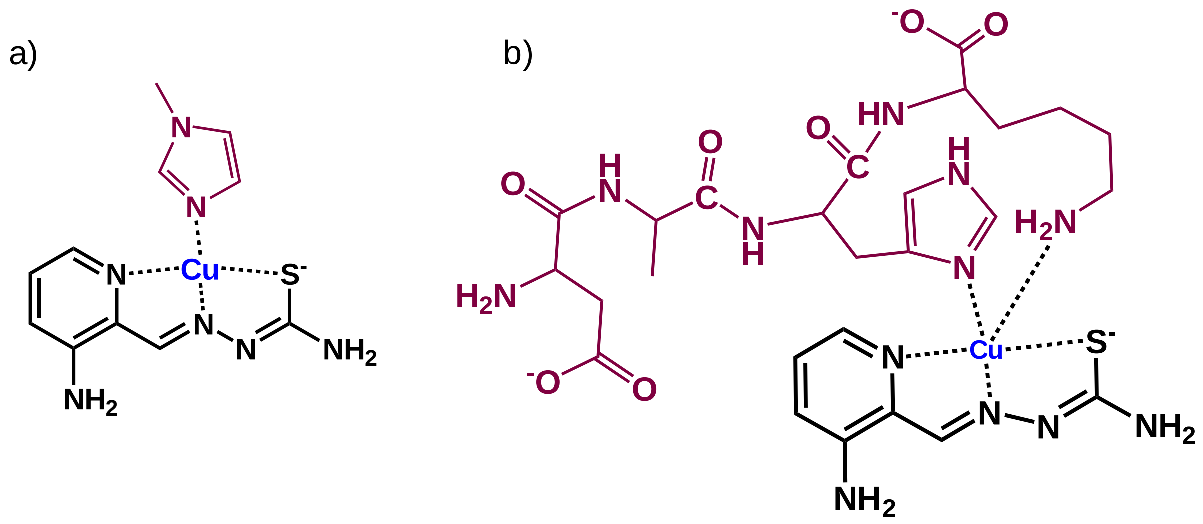


**Scheme S1.** Possible structures for the [CuAL] ternary complexes formed in the **a**) copper(II) – Triapine – mim and **b**) copper(II) – Triapine – DAHK systems.
